# Special Issue “Serotonin in Health and Diseases”

**DOI:** 10.3390/ijms27031211

**Published:** 2026-01-25

**Authors:** Elena E. Voronezhskaya

**Affiliations:** Koltsov Institute of Developmental Biology, Russian Academy of Sciences, Moscow 119334, Russia; elena.voronezhskaya@idbras.ru

Serotonin is most often mentioned in association with the brain, neuronal circuits, and behavior. Indeed, its role as a neurotransmitter regulating mood, cognition, and behavior has been extensively documented in a vast body of experimental and clinical research. However, accumulating evidence clearly demonstrates that serotonin (5-hydroxytryptamine, 5-HT) is far more than a classical neurotransmitter; it is an evolutionarily ancient biogenic amine, present across most metazoan phyla, emerging at the earliest stages of ontogenesis and accompanying an organism throughout its entire lifespan. In this broader framework, monoamines are not primarily considered as mediators of behavioral regulation in the mature nervous system, but rather as integrating components of organismal adaptation during development, whose modulation shapes cellular properties, tissue organization, and physiological set points, thereby giving rise to long-lasting and delayed phenotypic effects. Acting through a complex system comprising synthesis and degradation enzymes, vesicular and membrane transporters, and more than fourteen receptor subtypes, serotonin represents a versatile and highly plastic signaling system with organism-wide regulatory capacity [1]. Accordingly, this Editorial deliberately emphasizes the developmental, peripheral, and intergenerational aspects of serotonergic signaling, rather than providing a comprehensive overview of adult neuropsychiatric disorders.

The serotonergic system exhibits several organizational principles that distinguish it from classical neurotransmission. First, 5-HT-containing neurons constitute a relatively small population but exert a disproportionately broad influence via widespread projections and volume transmission. Second, 5-HT receptors and transporters are expressed in nearly all tissues, allowing cells positioned far from 5-HT release sites to respond to 5-HT. Third, all components of the system display pronounced developmental and environmental plasticity, with anatomical organization and molecular machinery being reshaped across life stages and in response to external conditions [2]. These features support the long-standing concept of 5-HT as a global integrative modulator rather than a purely local mediator [2,3,4,5].

Crucially, 5-HT and serotonergic signaling components appear long before nervous system formation, and 5-HT is detected in oocytes, zygotes, and early cleavage-stage embryos, where it contributes to fundamental processes of cell competence, early patterning, and morphogenetic movements [6,7,8,9,10]. As development proceeds, serotonergic signaling components are among the first neurochemical systems expressed in differentiating neurons, influencing neurogenesis, neuronal phenotype specification, and the maturation of endocrine axes [11,12,13,14,15]. From an evolutionary perspective, serotonin signaling predates neurons themselves and 5-HT represents an archetypical signaling molecule conserved across animal evolution [4,16,17,18].

Beyond the nervous system, the serotonergic system plays a dominant role in peripheral physiology. Peripheral serotonin signaling regulates cardiovascular and immune functions, energy metabolism, insulin secretion, hematopoiesis, and organ-specific physiology in tissues such as the gonads, lungs, and liver [19,20,21,22]. The remarkable functional diversity of 5-HT actions is enabled by the existence of at least fifteen receptor genes grouped into four major families [23], allowing simultaneous activation of multiple intracellular signaling cascades within the same tissue.

Importantly, contemporary studies have revised the classical endocrine view of peripheral 5-HT action. In addition to the circulating 5-HT pool, many organs possess local serotonergic systems in which serotonin-producing and serotonin-responding cells are spatially juxtaposed, enabling autocrine and paracrine regulation. Such local systems have been described in the pancreas, thymus, mammary gland, bone marrow, and adrenal glands [15,20,22,24]. Moreover, some systems utilize non-canonical modes of 5-HT action, including post-translational modification of intracellular proteins [10,25,26,27], thereby providing long-lasting and delayed effects. This organizational logic further reinforces the role of 5-HT as a context-dependent and environmentally sensitive signaling molecule with integrative properties.

Taken together, these properties place the serotonergic system at the core of developmental programming, where parental physiological state, environmental inputs, and early embryonic signaling converge to shape long-term neural, behavioral, and somatic outcomes. Within the framework of Developmental Origins of Health and Disease concept (DOHaD), serotonin signaling emerges as a key molecular axis linking parental condition to embryonic development and adult health trajectories. Its early appearance, systemic distribution, and lifelong effects on neuronal and neuroendocrine cell plasticity make it uniquely suited to mediate perinatal neurochemical programming and to translate transient developmental perturbations into persistent phenotypic effects.

In this context, the articles collected in this Special Issue can be viewed as complementary explorations of a single conceptual continuum: serotonin signaling as a parent–embryo–adult axis of intergenerational regulation. They address role of 5-HT as a transient but dominant placental signal, its sensitivity to maternal stress and inflammation, its capacity to mediate parental effects in invertebrates, and its long-term consequences for behavior, neural circuitry, and peripheral physiology. Together, these studies provide a coherent framework for understanding how serotonergic signaling integrates environmental information across generations and developmental stages, setting the stage for the detailed analysis of individual contributions presented below. 

A central theme emerging across several articles in this Special Issue is the role of the placenta in maintaining fetal 5-HT levels. Bondarenko et al. demonstrate that the placenta is the primary source of 5-HT for the mammalian embryo and that placental contributions exceed embryonic 5-HT synthesis during most developmental stages. Notably, inflammatory perturbations in the mother organism can significantly alter placental 5-HT levels. The authors provide experimental evidence that immunoglobulin administration can prevent such effects, and suggest a potential therapeutic approach [Contribution 1]. Complementing these findings, Melnikova et al. report that prenatal stress exerts a bidirectional influence on placental 5-HT synthesis: mild stress enhances, while moderate stress suppresses placental 5-HT production, leading to distinct behavioral phenotypes in the offspring [Contribution 2]. Collectively, these studies underscore the placenta as a dominant source of 5-HT during development through which dynamic changes in critical windows affects behavioral outcomes in offspring.

Moving earlier in ontogenesis, several contributions show that serotonin signaling is already active in the oocyte and preimplantation embryo, long before canonical neuronal circuits emerge. Frolova et al. combine transcriptomic and structural–functional analyses to demonstrate that mouse oocytes and cleavage-stage embryos contain 5-HT, express VMAT2 and multiple membrane receptors (5-HT_1B_, 5-HT_1D_, 5-HT_2B_, 5-HT_7_), and require vesicular transport for maintaining signaling integrity [Contribution 3]. Alyoshina et al. further refine this by demonstrating that SERT (membrane 5-HT transporter) activity in mouse oocytes correlates with follicular growth and oocyte maturity, linking 5-HT uptake capacity to granulosa proliferation and follicle size. Pharmacological inhibition of SERT with fluoxetine substantially reduces 5-HT content in developing oocytes [Contribution 4]. These results indicate that serotonin signaling serves as an important contributor to oocyte competence and early embryonic viability.

An evolutionary perspective is provided by Shestipalova et al., who examine parental serotonergic system modulation in the freshwater mollusk *Lymnaea stagnalis*. Their results demonstrate that parental 5-HT levels shape monoaminergic phenotypes in identified embryonic neurons, modulating embryonic locomotor behavior and nutrient consumption without affecting dopaminergic balance [Contribution 5]. This work highlights the conserved role of serotonin signaling in early neural development and demonstrates how neuronal phenotype can be altered through environmentally sensitive parental contributions.

Transitioning from early development to mature physiology, Sgambato provides a detailed review of the properties and therapeutic potential of the 5-HT_4_ receptor, emphasizing its conserved expression across species in the basal ganglia, cortex, hippocampus, amygdala, and peripheral organs such as the gastrointestinal tract [Contribution 6]. The review highlights the unique capacity of 5-HT_4_ to enhance acetylcholine and dopamine release, explaining its rapid antidepressant and pro-cognitive effects, as well as its regulatory role in feeding behavior and gastrointestinal function. These properties make 5-HT_4_ a promising target for a spectrum of neurological and systemic disorders.

Redina et al. extend this receptor-focused perspective by analyzing strain-specific transcriptomic profiles in the hypothalamus of hypertensive and normotensive rats, both under basal conditions and acute stress [Contribution 7]. Authors identify the differentially expressed genes associated with 5-HT synthesis, transport, and receptor-mediated signaling, with cross-talk involving dopaminergic, GABAergic, cholinergic, and glutamatergic pathways. Their findings underscore the integration of serotonergic signaling into broader neurochemical networks that respond dynamically to stress and genetic background. 

Finally, Sadykova et al. demonstrate the pathological consequences of serotonin signaling dysregulation in cardiovascular tissues. They report elevated SERT and 5-HT_2_A/5-HT_2_B receptor expression in the aorta and heart of immature LDL-receptor-deficient mice, correlated with early-stage atherosclerotic plaque formation [Contribution 8]. Their results implicate 5-HT as a mediator of vascular remodeling and lipid accumulation, suggesting that components of serotonin signaling could serve as potential biomarkers or therapeutic targets in both juvenile and adult cardiovascular disease.

Taken together, the articles in this Special Issue trace a continuous trajectory of serotonin signaling and the serotonergic system: beginning with 5-HT placental supply and oocyte signaling, progressing through the establishment of a neural phenotype, to reaching adult receptor-mediated physiology regulation. Looking forward, several promising directions arise from the studies of this Special Issue. First, the integration of developmental biology with transcriptomic, epigenetic, and functional imaging approaches provides a powerful framework for identifying critical windows during which 5-HT and serotonergic signaling perturbations exert long-lasting effects. Second, components of the serotonergic signaling—such as placental 5-HT synthesis enzymes, transporters, and receptor subtype expression patterns—emerge as potential biomarkers of developmental risk and resilience. Finally, receptor-specific and context-dependent modulation of serotonin signaling may offer novel preventive or therapeutic strategies for neurodevelopmental, metabolic, and cardiovascular disorders rooted in early-life programming.

The perspectives emerging from the studies presented in this Special Issue underscore 5-HT as a multifaceted coordinator of cellular communication whose modulation by environmental, genetic, or pathological factors has long-lasting implications for organismal health and disease. We hope that these insights will inspire readers to conduct further research, foster interdisciplinary collaboration, and help to identify strategies for harnessing the serotonergic system to improve health throughout the lifespan.
